# Mood Disorders in Mothers of Children on the Autism Spectrum Are Associated with Higher Functioning Autism

**DOI:** 10.1155/2012/435646

**Published:** 2012-08-15

**Authors:** Roma A. Vasa, Connie Anderson, Alison R. Marvin, Rebecca E. Rosenberg, J. Kiely Law, Julia Thorn, Geeta Sarphare, Paul A. Law

**Affiliations:** ^1^Department of Psychiatry, Kennedy Krieger Institute, Johns Hopkins University School of Medicine, 3901 Greenspring Avenue, Baltimore, MD 21211, USA; ^2^Department of Medical Informatics, Kennedy Krieger Institute, Baltimore, MD 21205, USA; ^3^Department of Pediatrics, Johns Hopkins University School of Medicine, Baltimore, MD 21287, USA

## Abstract

Mood disorders occur more frequently in family members of individuals with autism spectrum disorders (ASD) than in the general population. There may be associations between maternal mood disorder history patterns and specific ASD phenotypes. We therefore examined the relationship between maternal mood disorders and child autism spectrum disorders in 998 mother-child dyads enrolled in a national online autism registry and database. Mothers of children with ASD completed online questionnaires addressing their child's ASD as well as their own mood disorder history. In multivariate logistic regression models of ASD diagnoses, the odds of an Asperger disorder versus autistic disorder diagnosis were higher among those children whose mothers had a lifetime history of bipolar disorder (OR 2.11, CI 1.20, 3.69) or depression (OR 1.62, CI 1.19, 2.19). Further, maternal mood disorder onset before first pregnancy was associated with higher odds (OR 2.35, CI 1.48, 3.73) of an Asperger versus autism diagnosis among this sample of children with ASD. These data suggest that differences in maternal mood disorder history may be associated with ASD phenotype in offspring.

## 1. Introduction

A higher prevalence of depression and bipolar disorder has been consistently reported in family members of children with autism spectrum disorders (ASD) compared with family members of children with other types of disabilities [1–3]. Furthermore, a large population study found that parents of children with autism were more likely to have been diagnosed with and hospitalized for a psychiatric disorder than parents of children in the general population [4]. 

It has been suggested that a history of parental mood disorder may be more strongly associated with certain ASD phenotypes. DeLong [5] hypothesized that there may be two “taxa” of ASD, one that is higher functioning, with prominent anxiety, obsessiveness, mood disorder, and a family history of major mood disorder, and another that is lower functioning, with a history of language and learning disability and no family history of mood disorder. 

Two studies have found that prevalence, types, and patterns of familial mood disorders indeed vary by ASD phenotype. DeLong and Dwyer [6] were the first to report such an association in their exploration of the family history of 51 individuals with ASD and a wide range of cognitive abilities. Their data revealed higher rates of Asperger disorder (also known as Asperger syndrome) in families of subjects with a verbal IQ (VIQ) greater than or equal to 70 compared to those with lower VIQs (68% versus 8%). They also found higher rates of bipolar disorder in families with a history of Asperger disorder compared to families with no such history (6.1% versus 3.3%) as well as a higher incidence of bipolar disorder in the families of children with VIQ greater than or equal 70 (although the latter finding did not achieve statistical significance). In a subsequent cross-sectional clinic-based study of 122 children with ASD, Cohen and Tsiouris [7] found that recurrent maternal depression was associated with higher cognitive and adaptive functioning, increased behavior problems, and an internalizing behavioral style in offspring. All mothers with recurrent major depression reported onset of their mood disorder prior to having children, providing support for the idea that depression in mothers may result from genetic contributions rather than solely from caregiver stress [7]. The findings of both studies suggest that familial mood disorders may be more common among children with higher functioning ASD.

There is increasing acceptance that individuals with ASD at whatever level of functioning share essential autistic deficits, falling along a spectrum rather than representing distinct syndromes. However, it is also clear that children with an Asperger disorder (ASP) diagnosis represent a cognitively normal-to-gifted, verbal, and relatively more socially engaged group relative to those with an autism (AUT) diagnosis. Other distinguishing features of ASP include an all-consuming preoccupation with one or more topic areas, an “eccentric and one-sided social approach to others,” and higher verbal relative to nonverbal skills [8–11]. In contrast, children with AUT may or may not have intellectual disability, delayed language, an “aloof” or “passive” style of interacting, and weaker verbal compared to nonverbal skills [8]. As a result of these differentiating clinical features, both researchers and clinicians use the ASP category (e.g., [12–15]) alongside established and anticipated (i.e., DSM-V) classification schemes.

 An association between maternal mood disorders and a higher functioning ASD phenotype, such as ASP, could help identify a subtype of ASD that is genetically and neurobiologically associated with maternal mood disorders. Previous smaller studies on this topic lacked power to evaluate and adjust for potential confounders or to include a significant number of participants who were positive for ASP, maternal bipolar disorder, or maternal depression. Therefore, in a large sample of 998 US mother-child dyads participating in an online autism research project, we examined the potential association between a lifetime history of maternal mood disorder and a high functioning phenotype of ASD in offspring. Using the ASP diagnosis as a proxy for a high functioning phenotype of ASD, we hypothesized the following.

1. Among mothers of children with ASD, those with a history of mood disorder will be more likely to have a child with ASP than AUT.
2. Among mothers of children with ASD who report a lifetime history of mood disorder, those who experienced their first mood disorder episode before their first pregnancy will be more likely to have a child with ASP than AUT.

## 2. Methods

### 2.1. Design and Participants

This study was conducted through the Interactive Autism Network (IAN), an online US based research registry and database launched in April 2007. IAN participants complete online questionnaires on a variety of topics to create a very large autism-focused dataset. IAN is open to all families living in the United States with at least one child with an ASD whose diagnosis was established by a professional in the community. More than 14,000 children younger than age 18 with ASD and their immediate family members are enrolled.

The Johns Hopkins Medicine Institutional Review Board approved all study procedures, and electronic consent was elicited from participating families. After enrollment, parents completed registration materials and were then invited to complete several questionnaires. These questionnaires were collaboratively developed by the IAN research team and members of the IAN Science Advisory Committee, subsequently piloted with families, and revised as needed. Established instruments such as the Social Responsiveness Scale (see below) [16] were also completed online. Analyses for the current study were based on data from questionnaires focusing on child ASD diagnosis, mother demographic and medical history, and maternal mood disorder history (MatMoodQ) completed between April 2007 and November 2008. IAN questionnaires are available at http://dashboard.ianexchange.org/DataExplorer.aspx.

Our initial sample consisted of 5,714 mother-child dyads in which

- the child was the eldest with ASD in the family, was a biological child under the age of 18 years upon launch of the MatMoodQ, did not have an ASD diagnosis of childhood disintegrative disorder or “recovered from ASD,” and had no history of Fragile X or Tuberous Sclerosis;
- the mother had provided demographic information and completed questionnaires focused on child ASD diagnosis and maternal medical history.

**Figure 1:**
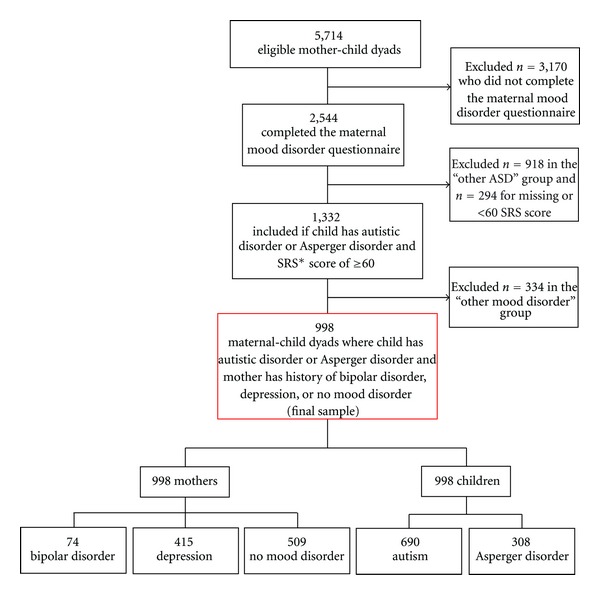
Participant eligibility diagram among children with autism spectrum disorder (ASD) and their biological mothers.

The derivation of the study sample is illustrated in Figure 1. Of the 5,714 eligible mothers, 2,544 (44.5%) completed the MatMoodQ. There was no difference between responders and nonresponders in terms of race, ethnicity, urbanicity, or child gender. Differences in mean maternal and mean child age at the time of MatMoodQ completion were statistically but not clinically significant between the two groups and are considered an artifact of the large sample size (effect sizes of .046 and .029 for maternal and child age, resp.). College educated mothers were more likely to participate (52.6% versus 46.9%, *P* < .001). Nonresponders were less likely to have completed the online SRS, but the difference in mean SRS T-scores for the two groups was not significant. Responders and non-responders did not differ in their responses to questions about mood disorder history on a basic medical history questionnaire. Specifically, nearly half of MatMoodQ responders (48.4%) and non-responders (46.4%) had reported a history of being “diagnosed with or treated for depression” (*P* = .158), while 5.0% of responders and 4.9% of non-responders said they had “been diagnosed with or treated for bipolar disorder” (*P* = .854).

### 2.2. Measures

#### 2.2.1. Child ASD Subtype (Dependent Variable)

The child ASD questionnaire solicited information about ASD diagnosis and developmental history. Response choices for ASD diagnosis included autism, Asperger disorder, pervasive developmental disorder not otherwise specified (PDD NOS), pervasive developmental disorder (PDD), and ASD. Response choices for the type of professional who had established the child's ASD diagnosis included a pediatrician, primary care doctor, developmental pediatrician, psychiatrist, clinical psychologist, neurologist, team of health professionals, team of professionals in a school system, and speech and language pathologist.

The Autism Diagnostic Interview-Revised (ADI-R) is a 93-item interview that the clinician administers to the parent or caregiver in order to assess for the presence of an ASD in the child. In one study of 107 children participating in the IAN registry, parent report of professionally diagnosed ASD was confirmed by developmental history using the ADI-R [17] in 99% of cases and by both ADI-R and direct observational assessment in 93% of cases [18]. Similarly, 98% of 116 IAN families participating in a second study were able to provide documentation verifying a professionally diagnosed ASD [19].

For the current analysis, children were sorted into one of three ASD categories: autism (AUT), Asperger disorder (ASP), or “Other ASD.” The “Other ASD” group (*n* = 918) was comprised of those with less well-defined diagnoses (PDD-NOS, PDD, or ASD), who were therefore excluded from further analysis because they comprised a more clinically heterogeneous group of children compared with those in the AUT and ASP groups [8, 20]. The Social Responsiveness Scale (SRS), a 65-item questionnaire completed by a caregiver or teacher to assess social awareness, social cognition, social motivation, social communication, and autistic mannerisms in a child, was then used to validate the parent-reported ASD diagnosis. As an SRS T-score of 60 or higher reflects social disability and probable ASD [16], we excluded 294 of the remaining children with AUT or ASP for whom SRS T-scores were unavailable (*n* = 245) or were less than 60 (*n* = 49) for an intermediate sample size of 1,332 (see Figure 1).

#### 2.2.2. Maternal Mood Disorder Status (Primary Independent Variable)

The maternal mood disorder questionnaire elicited data on lifetime history of professional- and self-diagnosed mood disorder as well as associated clinical features, such as when the mood disorder began (before or after having children) and its clinical severity as reflected by psychiatric hospitalization, suicidality, and number of recurrent episodes of depression. Participants could report none, one, or more than one of the following diagnoses: bipolar disorder, cyclothymia, major depressive disorder (MDD), seasonal affective disorder, dysthymia, postpartum depression, other “hormonal” depression (premenstrual syndrome, premenstrual dysphoric disorder, or depression related to menopause), or “some kind of depression” (refers to mothers who did not know their specific diagnosis). The overall approach was similar to the clinically validated method for collecting child ASD diagnosis online described above.

Mothers were categorized into four groups based on mood disorder status: no history of mood disorder (*n* = 509), bipolar disorder (BP) diagnosed by a professional (*n* = 74), depression (DEP) diagnosed by a professional (*n *= 415), and “other mood disorder” (*n* = 334). Those who reported both BP and one or more depressive diagnoses were included in the BP group (i.e., BP and DEP were mutually exclusive).

The “other mood disorder” group (*n* = 334) consisted of mothers who reported any of the following: cyclothymia, a nonspecific mood disorder, not knowing which professional established their mood disorder diagnosis, self-diagnosis of their mood disorder, or receiving treatment for a mood disorder without a formal diagnosis. This group of mothers with indeterminate mood disorder status was excluded from our intermediate sample of 1,332 mother-child dyads for a final study sample of 998 mother-child dyads with the most well-characterized ASDs and mood disorders (see Figure 1).

### 2.3. Other Variables

Race, ethnicity, mother and child date of birth, and child gender were ascertained from information shared by families upon IAN registration. Child developmental history, including language acquisition, most recent IQ score, and history of intellectual disability, was obtained from the child ASD questionnaire. Data on IQ and/or intellectual disability status and language development were reported for only a limited group of children (see Table 1).

Urbanicity was analyzed using the six-level 2006 NCHS Urban-Rural (NCHSUR) Classification Scheme for Counties [21]. For this study, we created 4 categories from the 6-item NCHSUR scale: large city (large central metro), suburban (large fringe metro), small/medium city (combines medium metro and small metro), and rural (combines micropolitan and noncore).

### 2.4. Data Analyses

All questionnaire data were maintained in the Internet Mediated Research System (IMRS) database (MDLogix, Baltimore, MD, USA). Analyses were performed using STATA 11.1 (College Station, TX, USA) on the live database. Fisher's exact tests and *t*-tests were used to compare demographic and clinical characteristics between children with AUT and ASP.

Multivariate logistic regression analysis was performed to estimate whether maternal BP (presence or absence) or DEP (presence or absence) was associated with a greater likelihood of having a child with an ASD diagnosis of ASP compared to AUT. The model included maternal mood disorder variables and *a priori* covariates such as child gender, maternal race, and maternal education level. Child and maternal age at the time the MatMoodQ was completed were also included as *a priori* covariates to account for the older mean age of the children with ASP and their mothers, and hence longer exposure time to develop a mood disorder. Urbanicity was not significant on bivariate analysis and was therefore not included. A *P* < .05 was considered significant for all analyses.

## 3. Results

### 3.1. Sample Characteristics

Table 1 presents the demographic and clinical characteristics of the mothers and their children. Compared to mothers of children with AUT, mothers of children with ASP were older, more likely to be white, less likely to be Hispanic, and more likely to have received a mood disorder diagnosis. Children with ASP were, as a group, older than children with AUT. There were also more males in the ASP group although this result was not statistically significant (*P* = .052).

Clinical characteristics of the 489 mothers with a professionally diagnosed mood disorder are presented in Table 2. Mothers with mood disorders, particularly BP, had high rates of depression recurrence. Most mothers with BP and DEP had the first onset of their mood disorder prior to their first pregnancy.

### 3.2. Multivariate Logistic Regression Models

We employed multivariate logistic regression to test the association of parent-reported, professionally-diagnosed lifetime history of maternal mood disorder with child ASP over AUT diagnosis, by specific mood disorder, as shown in Table 3. Maternal history of BP was associated with significantly higher adjusted odds of having an affected child with ASP compared with AUT (OR 2.11, CI 1.20, 3.69). Similarly, maternal DEP was associated with a significantly greater likelihood of ASP versus AUT (OR 1.62, CI 1.19, 2.19). The mean variance inflation factor (VIF) for the model was 1.23 indicating minimal collinearity.

Next, employing the same statistical method, we tested our second hypothesis to find out whether the adjusted odds of having a child with ASP versus AUT would be greater for mothers who first experienced their mood disorder prior to having children compared to mothers who first experienced their mood disorder after having children. As shown in Table 4, mothers with prepregnancy mood disorder were 2.35-times more likely (CI 1.48, 3.73) to have a child with ASP versus AUT. The mean variance inflation factor (VIF) for the model was 1.28, again indicating minimal collinearity.

## 4. Discussion

The results from this study add to a small body of literature demonstrating an association between maternal mood disorders and a higher functioning phenotype of ASD. We found that a lifetime history of maternal BP or DEP each was more strongly associated with having a child with ASP compared to AUT after controlling for relevant maternal and child covariates. The data suggest that this association was stronger for maternal BP than DEP. Furthermore, having a child with ASP versus AUT was more likely if mothers had the first onset of their mood disorder prior to having any children. These findings are consistent with those of DeLong and Dwyer [6] who found higher proportions of bipolar disorder in the first and second degree relatives of probands with a family history of ASP [6], and also with those of Cohen and Tsiouris [7] who found associations between maternal depression and a high functioning ASD phenotype in offspring. Our data extend prior work by examining these associations using an internet-based study design that collected maternal self-report data on community-based clinician diagnoses of maternal mood disorder and child ASD.

Why might maternal mood disorders be associated with a higher functioning phenotype in offspring with ASD? One hypothesis is that psychosocial factors may play a role; mothers of children with ASP may experience a different type of caregiving stress than mothers of children with AUT. For example, children with ASP typically receive their diagnosis later than children with AUT [22, 23]. It is possible that this prolonged diagnostic odyssey, combined with the baffling mixture of strengths and deficits displayed by these children, results in stress different from that experienced by families of children with a more severe, but less ambiguous, status. In addition, children with ASP have high rates of psychopathology, which can exacerbate caregiver stress [20, 24, 25] and are also most likely to be educated in typical classrooms where they may face difficult social challenges on a daily basis. However, it is difficult to imagine that mothers of children who are higher functioning experience greater overall stress than mothers of more severely affected children. Moreover, our data as well as the findings of other studies indicate that the likely attribution of maternal mood disorders to psychosocial stress is low because most mothers were diagnosed with a mood disorder prior to the birth of their child [2, 3, 7, 26, 27]. Therefore, psychosocial stress is unlikely the sole determinant underlying this association.

Alternately, there may be a genetic etiology underlying the association between maternal mood disorders and the ASP, rather than AUT, subtype of ASD. As previously mentioned, Delong [28] proposed two “taxa” of ASD: one that is higher functioning, associated with comorbid anxiety and mood disorders, and linked to a family history of mood disorder (ASP-related), and another that is lower functioning and not linked to a family history of mood disorder (AUT-related). It is possible that shared genetic susceptibilities may give rise to both mood disorders in the mother and the ASP phenotype in offspring. Current research suggests that different genetic etiologies may give rise to different subtypes of autism [29]. For example, in a sample of 119 males with ASD, autism severity in terms of aggression, fears, and rituals was modified by the mother's monoamine oxidase A (MAOA) genotype [30]. There is also evidence of differential heritability for different ASDs and ASD-associated traits, as illustrated by a study of 277 twin pairs where at least one twin had an ASD and in which there was higher psychiatric comorbidity, functioning level, and Asperger syndrome concordance among affected monozygotic versus dizygotic twins [31]. An alternative genetic explanation for the current findings is that maternal mood disorders may “exert a protective effect on cognitive and adaptive functioning” [7]. According to this idea, in offspring predisposed to developing an ASD, the presence of a maternal mood disorder exposure modifies expression into ASP rather than AUT. Our finding that onset of mood disorder before having children is associated with having a child with ASP, as opposed to AUT, potentially provides support for either of these hypotheses, both of which merit further investigation.

It is important to note that even mothers of children with AUT had high rates of mood disorder, and that a significant number of all mothers with BP or DEP reported inpatient psychiatric hospitalization, suicidality, and recurrent episodes of depression. Clinicians caring for children with ASD are therefore encouraged to consider screening for mood disorders in the mother and to be ready to offer appropriate treatment resources and referrals.

This study has several unique strengths, most notably its large sample size, which enables research on patterns of disease that may be difficult to appreciate in smaller samples. The large sample size also allows for adjustment of potentially confounding variables such as child gender, age, and maternal education level. An additional strength is that health information was collected via the Internet. Current research supports web-based surveys of medical information as a reliable means of data collection [32]. This has been demonstrated by a similar registry for a disorder related to autism, Rett syndrome [33], and in other studies using IAN data [34]. Advantages of internet-based studies include low cost, decreased response burden, greater ease of participation, access to families from rural areas, and anonymity [35, 36]. Internet usage is high among individuals of parenting age in the US (79% to 87% in 18 to 54 years old; [37]). 

This study has several limitations. First, ASD and mood disorder diagnoses were based on maternal self-report of a clinician diagnosis rather than an in-clinic assessment conducted as part of the study. However, our research was intentionally designed to examine community-based patterns of disease in children with ASD using a self-report data collection method. IAN data show that this method is reliable and valid for ascertaining community-assigned ASD status in participating children [18, 19]. An IAN validation study of the maternal mood disorder diagnosis has not been conducted. The current study, however, applied stringent self-report criteria by only including mothers who reported a diagnosis of BP or DEP made by a medical or mental health professional and eliminated the 25% of mothers reporting a less well-characterized mood disorder (e.g., self-diagnosed, treated but not formally diagnosed). Some diagnostic misclassifications may have occurred, but these are likely to be overcome by the study's large sample size. Another point to note is that the intent of this study was not to approximate a generalizable population prevalence of actual maternal mood disorders in families of children with ASD, but to determine relative frequencies of illness within the IAN population and examine associations with child phenotype.

Our sample of families may not reflect all US households. The mothers in our study were primarily white, non-Hispanic, highly educated, and over the age of 30 years. However, this demographic profile is similar to that of subjects in other online and clinic-based studies [38–42], and there is evidence that web-based samples may actually be more representative of the general population than samples in center-based studies [32, 43]. Further, although there is a risk of self-selection bias, with affected mothers more likely to complete a maternal mood disorder questionnaire, our findings are based on between-group comparisons, which are largely resistant to this type of selection bias. In addition, when examining frequency of self-reported BP or DEP on a general medical history survey, we found no difference in responses between MatMoodQ responders and nonresponders. In other words, those who had earlier reported a history of BP or DEP were no more likely to take the MatMoodQ than those who had not. 

## 5. Conclusion

In summary, data from this large community and internet-based study suggest that mothers of children with ASD who have a lifetime history of BP or DEP are more likely to have a child with the ASP versus AUT phenotype. Similarly, among mothers of children with ASD who report a lifetime history of mood disorder, those with prepregnancy onset are more likely to have a child with ASP than AUT. While the precise etiology of this link remains unclear, these findings are consistent with prior studies and merit further exploration.

## Figures and Tables

**Table 1:** Demographic and clinical characteristics of the final study sample of maternal-child dyads after all exclusions were applied (*n* = 998).

	Autistic disorder	Asperger disorder	Fisher's exact/paired *t*-test *P* value
	*n* = 690 (69.8%)	*n* = 308 (31.2%)	
Mean maternal age (SD)^∗^	38.6 (6.7)	41.7 (6.5)	<.001
Maternal race (% white)	632 (91.6)	296 (96.1)	<.05
Maternal ethnicity (% Hispanic)	47 (6.8)	10 (3.3)	<.05
Maternal education (% college degree)	344 (49.9)	173 (56.2)	ns
Urbanicity^∗∗^			
Large city (%)	157 (22.8)	66 (21.4)	ns
Suburban (%)	224 (32.5)	105 (34.1)	
Small/med city (%)	210 (30.4)	92 (29.9)	
Rural (%)	99 (14.4)	45 (14.6)	
Maternal mood disorder diagnosis (%)			
No mood	377 (54.6)	132 (42.9)	.001
Bipolar disorder	43 (6.2)	31 (10.1)	
Depression	270 (39.1)	145 (47.1)	
Mean child age (SD)^∗^	8.3 (3.8)	11.1 (3.6)	<.001
Child gender (% female)	124 (18.0)	40 (13.0)	.052
Intellectual level^∗∗∗^			
IQ ≥ 116 (%)	23 (11.1)	108 (50.5)	<.001
Phase speech by age 3 years^∗∗∗∗^	175 (26.9)	220 (74.1)	<.001

^∗^Mean maternal and child age at the time the maternal mood disorder questionnaire was completed.

^∗∗^Based on NCHS Urban-Rural (NCHSUR) Classification Scheme for Counties [21].

^∗∗∗^ *n* = 422 (208 with AUT; 214 with ASP).

^∗∗∗∗^ *n* = 948 (651 with AUT; 297 with ASP).

**Table 2:** Self-reported clinical characteristics of mothers of children with an autism spectrum disorder and reported professional diagnosis of mood disorders (*n* = 489).

	Bipolar disorder	Depression	Fisher's exact/paired *t*-test *P* value
	*n* = 74	*n* = 415	
Type of professional establishing diagnosis			
Psychiatrist	51 (68.9)	94 (22.7)	<.001
Psychologist	16 (21.6)	67 (16.1)	
Nonpsychiatrist physician	6 (8.1)	219 (52.8)	
Therapist	1 (1.4)	35 (8.4)	
Severity of mood disorder (%)			
Inpatient psychiatric hospitalization	27 (37.0)	53 (12.9)	<.001
Considered/attempted suicide	53 (71.6)	232 (56.2)	<.05
3 or more depressive episodes	69 (95.8)	328 (80.2)	<.01
Onset of mood disorder before having children (%)	63 (86.3)	266 (64.3)	<.001

**Table 3:** Multivariate logistic regression analysis of Asperger syndrome (ASP) versus autistic disorder (AUT) phenotype among children with ASD by presence of professionally diagnosed maternal mood disorder (*n* = 998 mother-child dyads).

Independent variables	Adjusted odds ratios
	ASP versus AUT (95% CI)
Maternal bipolar disorder	2.11^∗^ (1.20, 3.69)
Maternal depression	1.62^∗^ (1.19, 2.19)
Maternal age	1.00 (0.98, 1.03)
Maternal race (ref = nonwhite)	0.63 (0.31, 1.26)
Maternal ethnicity (ref = Hispanic)	0.56 (0.26, 1.20)
Maternal education (ref = college degree)	1.26 (0.93, 1.70)
Child age	1.20^∗∗^ (1.15, 1.26)
Child gender (ref = female)	0.73 (0.48, 1.11)

^∗^ *P* < .05, ^∗∗^ *P* < .001.

**Table 4:** Multivariate logistic regression analysis of Asperger syndrome (ASP) versus autistic disorder (AUT) phenotype among children with an ASD by timing of onset of maternal mood disorder among affected mothers (*n* = 487).

Independent variables	Adjusted odds ratios
	ASP versus AUT (95% CI)
Onset of mood disorder	2.35^∗∗^ (1.48, 3.73)
before having children	
Maternal age	1.01 (0.97, 1.05)
Maternal race (nonwhite)	1.76 (0.57, 5.47)
Maternal ethnicity (Hispanic)	0.33 (0.09, 1.16)
Maternal education (college degree)	1.17 (0.76, 1.78)
Child age	1.25^∗∗^ (1.17, 1.34)
Child gender (female)	0.99 (0.55, 1.76)

*P < .05, **P < .001.

## Abbreviations

BP: Bipolar disorder
DEP: Depression
ASP: Asperger syndrome
AUT: Autistic disorder.
